# Quality of Life Among Breast Cancer Patients in Mures County, Romania: A Cross-Sectional Study

**DOI:** 10.7759/cureus.65870

**Published:** 2024-07-31

**Authors:** Oltean Andra, Cezara Pintea, Andrei Manea, Aurel Nirestean, Raluca Niculescu, Mircea Gîrbovan, Strete Elena-Gabriela

**Affiliations:** 1 Department of Psychiatry, Emergency Clinical County Hospital of Târgu Mureș, Târgu Mureș, ROU; 2 Faculty of Pharmacy, George Emil Palade University of Medicine, Pharmacy, Science, and Technology of Târgu Mureș, Târgu Mureș, ROU; 3 Department of Radiology, Emergency Clinical County Hospital of Târgu Mureș, Târgu Mureș, ROU; 4 Department of Psychiatry, George Emil Palade University of Medicine, Pharmacy, Science, and Technology of Târgu Mureș, Târgu Mureș, ROU; 5 Department of Pathophysiology, George Emil Palade University of Medicine, Pharmacy, Science, and Technology of Târgu Mureș, Targu Mures, ROU; 6 Department of Urology, Emergency Clinical County Hospital of Târgu Mureș, Târgu Mureș, ROU

**Keywords:** disabilities, psychiatric disorders, personality, life quality, breast cancer

## Abstract

Background and aim

Psychiatric pathology does not always start on its own but may be conditioned or triggered by a comorbidity with a high impact on the human psyche. When there are comorbidities, psychiatric pathology can occur due to the high diagnostic burden. Our study aims to find out if there is a correlation between the diagnosis of breast cancer and its severity, and psychiatric symptoms such as depressive mood, atypical anxiety, or even autolytic ideation that directly influence the quality of life of patients.

Materials and methods

The study is a prospective, cross-sectional, single-center study carried out between December 2023 and June 2024 at the Mureș County Clinical Hospital in Romania. The sample population had to be at least 18 years old and had to be diagnosed with breast cancer recently. We applied two tests, WHODAS 2 (World Health Organization's Disability Assessment Schedule 2.0) and level 1 (level 1 of cross-sectional measurements of symptoms), to be able to measure and aid assessment of mental health domains that are important across psychiatric diagnoses and also the degree of disability triggered by breast cancer. The statistical analysis included descriptive statistics and interferential statistics. Statistical tests, such as Shapiro-Wilk, Kruskal-Wallis, and Mann-Whitney U tests with Bonferroni correction tests, were used. The p-value was set to 0.05 with a confidence interval (CI) of 95%.

Results

The study included 120 women diagnosed with breast cancer, with a mean age of 56.64 ± 9.46 years. Regarding the severity of the diagnosis, 44 women (36.66%) had non-invasive cancer, 58 (48.33%) had invasive cancer, and 18 (15%) had metastases. There was a statistically significant difference between three of the five selected level 1 domains across cancer types. The WHODAS 2.0 disability scores showed a significant difference between groups (p < 0.001). Subjects with non-invasive cancer had the lowest WHODAS 2.0 score, followed by the invasive group, while metastases had the highest score.

Conclusions

Following the application of the two tests, level 1 and WHODAS 2.0, to our group of subjects, statistically significant differences were observed between the three categories of subjects. The degree of disability and the occurrence of psychological symptoms differed according to the severity of breast cancer. Adapting to the status of an oncological patient entails multiple changes from a psycho-emotional, social, occupational, and professional point of view. Although the most recent medications prolong survival, a holistic approach that considers psychological aspects can improve patients' long-term results.

## Introduction

In delimiting the concept of normality, the notion of mental health comes into play, defined, as a rule, by the capacity for awareness, acceptance, and correctness in the way the individual conceives himself; the control of the environment and the adequacy in the way to face the demands of life; integration and unity of personality; autonomy and self-confidence; realistic perception and social sensitivity; and the continuity of personal development toward self-actualization [1,2].

Psychiatric pathology is associated in countless cases with somatic conditions that interfere with the dynamics of psychopathological episodes, with dimensions and personality traits; therefore, the onset, evolution, and prognosis of psychopathological diversities can be somatically conditioned. Breast cancer is the most frequently diagnosed cancer in women [3]. It is a complex disease that is associated with serious damage to one’s image, with deep anguish, significantly affecting the quality of life of patients. Stigmatization, routine, and fear are elements often associated with this diagnosis [4].

The attitude of patients toward this disabling disease is closely dependent on both mental balance and individual personality traits that are directly involved in disease progression, therapeutic response, and subsequent psychosocial rehabilitation [5]. The impact of the diagnosis of breast cancer most of the time brings irreversible damage to the psyche, which is why it is mandatory to do a psychiatric examination right from the beginning [2,6]. After that, fear of death, hopelessness, changes in life, and poor quality of life may give negative perceptions in breast cancer patients [6].

According to studies, approximately half of the patients diagnosed with breast cancer meet the criteria for a psychiatric diagnosis, the symptoms observed in these patients being manifestations of the pain caused by the disease or reactions to treatment or diagnosis [7]. Oncology patients associated with severe pain have a double risk of developing psychopathological episodes compared to those with less intense or even absent pain. At the same time, somatic and psychiatric comorbidity considerably increases the risk of suicide in patients associated with depression [8].

The purpose of investigations and treatment is not only the remission of psychiatric symptoms but also the improvement of the quality of life of cancer patients. Reactive symptoms to the “new” situation, to the condition of a sick person, further complicate the clinical picture of the underlying disease, and in the case of the perspective of surgical intervention, the psychopathological picture, predominantly anxiety, is exacerbated by the unpredictability of the operative act [9].

Depression occurring at this level is defined as a mood disorder associated with profound sadness, which is associated with behavioral helplessness. There are progressive symptoms such as asthenia, fatigue, a decrease in overall performance, even to the point of hypotonia, and a lack of interest in daily routines (biological, food, clothing, professional, and social needs). These symptoms are exacerbated by the micromanical idea of self-devaluation, uselessness, and incurability that is associated in most cases [10,11].

Somatic and psychological comorbidity also increases autolytic risk. If we consider suicide as a defensive reaction or an act of cowardice in the face of seemingly insurmountable existential difficulties, concerns about death can intersect the individual lives of both normal and pathologically structured individuals [12]. “The desire for meaning” [13] is the basic element of individual existence and is closely related to the person’s attitude toward himself. Loss of motivations that give meaning to life leads to autolytic concerns [14].

The appearance of a disabling disease produces a series of disturbances that propagate in a chain in the family and professional environment of the patient (the micro- and macro-group to which she belongs). The establishment of some rules, whether legally regulated or not, requires compliance from both the patient and their social groups of belonging [15].

Therefore, the main aim of this study is to confirm or deny the occurrence of psychiatric symptomatology in oncologic patients and also to determine the degree of disability of patients post-diagnosis. This is done to improve the quality of life of patients through early therapeutic intervention.

World Health Organization Disability Assessment Schedule 2.0 (WHODAS 2.0)

The biopsychosocial model entitled International Classification of Functioning, Disability, and Health was created by the World Health Organization (WHO), to be able to classify and describe the health of patients in terms of functioning, activities, and environmental factors. As it is difficult to apply in clinical practice, the WHO is developing a new assessment tool, Disability Assessment Schedule - WHODAS 2.0. This tool measures the disability and functionality of individuals, which is proposed by the Diagnostic and Statistical Manual of Mental Disorders, Fifth Edition (DSM-5), as a standard tool for functionality [16]. Based on studies, the validation hypotheses of WHODAS 2.0 are as follows: (1) six disability factors; (2) the scores will be related to depression levels [17]; illness severity, social and occupational functioning [18], and health-related quality of life; and (3) the degree of disability is higher to those patients who are not working [19].

WHODAS 2.0 assesses the degree of disability in six domains: understanding, communication, activities in the immediate environment, personal autonomy, interpersonal relationships, and activities of daily living. Response options go from 1 (no difficulty) to 5 (extremely difficult or cannot do) [20].

Level 1 test (level 1 of cross-sectional symptom measurements)

The DSM-5 level 1 cross-symptom measure is a self-report measure that assesses domains of mental health that are relevant to all psychiatric diagnoses. It is designed to help specialists provide additional information about domains that may have an impact on treatment and prognosis.

The adult version of the questionnaire consists of 23 questions that assess 13 psychiatric domains: depression, anger, mania, anxiety, somatic symptoms, suicidal ideation, psychotic ideation, sleep problems, memory, recurrent thoughts and behaviors, dissociation, personality functioning, and consumption of substances [20].

## Materials and methods

A cross-sectional study was conceptualized at the Mureș County Clinical Hospital in Romania. The inclusion criteria consisted of diagnosis of breast cancer, age above 18 years, and female patients. The exclusion criteria consisted of the presence of other comorbidities associated with a major impact on psychism and former diagnosis of personality disorders or chronic psychiatric diseases. Afterward, we divided them into three categories (non-invasive cancer, invasive cancer, and cancer with metastases) based on the biopsy report. We aimed to find how much the patient was affected by certain symptoms corresponding to each individual item over the last two weeks.

The subjects diagnosed with breast cancer selected by the inclusion and exclusion criteria were informed, by word and in the introduction of the questionnaire, that the data being collected were anonymous and would be used for scientific purposes. The respondents’ choice to participate in the study was not constrained, and their participation did not impact the quality of medical care received at the clinic. The Ethics Commission of the "George Emil Palade" University of Medicine, Pharmacy, Science, and Technology of Târgu Mureş approved the research with decision no. 1853/13.09.2022. Before participation in the study, all patients signed an informed consent form.

Participants and procedure

The study sample includes a total number of 120 subjects diagnosed with breast cancer at the Mureș County Clinical Hospital. The subjects originated from both rural and urban areas. We formed three groups of subjects based on the severity of the diagnosis (non-invasive cancer, invasive cancer, and cancer with metastases). After the subjects talked to the doctor, such as the treatment options, the life expectancy, and the quality of life after the treatment, we waited three more weeks after the diagnosis and doctor-patient discussion to allow them to comprehend the diagnosis and seek more information. After this period, we sent DSM level 1 and WHODAS 2.0 questionnaires for an online self-assessment.

All subjects signed an informed consent form before completing the DSM level 1 and WHODAS 2.0 and having their data included in the database.

Measures

To evaluate the subjects, we applied the World Health Organization Disability Assessment Inventory WHODAS 2.0 and the level 1 test (level 1 of cross-sectional symptom measures that assess mental health domains important for all psychiatric diagnoses). The tests were in Romanian language based on the official translation of the guides. These two tests were used to evaluate the quality of life of the patients after receiving the breast cancer diagnosis, to develop evidence of individual vulnerability from a psychopathological point of view, and to assess the capacity for resilience to diversify and increase the complexity and efficiency of therapeutic strategies.

We used five of the most affected domains of the DSM level 1 test: domain I - depression, domain IV - anxiety, domain V - somatic symptoms, domain VI - suicidal ideation, and domain VIII - sleep disorders. The questions in level 1 covered symptoms in the past two weeks. Patients are asked to respond on a five-point scale to record responses about the intensity and the frequency of the symptoms over the previous two weeks as follows: 0 = none/not at all; 1 = slight/rare, less than a day or two; 2 = mild/several days; 3 = moderate/more than half the days; and 4 = severe/nearly every day.

WHODAS 2.0 lists 12 activities of daily living and asks patients to rate the level of difficulty of the tasks performed in the last 30 days. A five-point rating scale is used (none = 0, mild = 1, moderate = 2, severe = 3, and extreme/cannot do = 4).

Statistical analysis

Statistical analysis was performed using the IBM SPSS v26 licensed software (IBM Corp., Armonk, NY), while the data manipulation was performed using Microsoft Excel 365 (Microsoft Corp., Redmond, Washington). The p-value was set to 0.05 with a confidence interval (CI) of 95%. The statistical analysis comprised descriptive statistics (mean, median, standard deviation, minimum, and maximum) and inferential statistics. The distribution of the data series was assessed using the Shapiro-Wilk test. We used the Kruskal-Wallis test to compare the numeric variables between the three groups. Afterward, we used the Mann-Whitney U test with Bonferroni correction to compare the variables pairwise.

## Results

All the subjects were female, originating both from the urban and the rural areas. Most of them originated from the urban environment (74 subjects, 61.66%), while 46 (38.33%) subjects originated from the countryside. The average age of the group was 56.64 ± 9.46 years. We also collected data about their workplace and grouped the professions into six major groups as follows: administration and support (27 subjects, 22.50%), healthcare (28 subjects, 23.33%), commerce and services (14 subjects, 11.66%), agriculture and food (10 subjects, 8.33%), education (9 subjects, 7.50%), and retiree (12 subjects, 10%), while 20 (16.66%) subjects were unemployed at the time of the study. Regarding the gravity of the diagnosis, 44 (36.66%) subjects had non-invasive cancer, 58 (48.33%) subjects had invasive cancer, and 18 (15%) subjects had metastases. All these demographic characteristics are summarized in Table 1.

**Table 1:** Demographic characteristics of the subjects M: Mean; SD: Standard deviation; n: Number of subjects.

Sample characteristics	N = 120
Age range, M (SD)	56.64 (9.46)
Living environment, n (%)	
Urban	74 (61.66%)
Rural	46 (38.33%)
Workplace, n (%)	
Administration and support	27 (22.50%)
Healthcare	28 (23.33%)
Commerce and services	14 (11.66%)
Agriculture and food	10 (8.33%)
Education	9 (7.50%)
Retiree	12 (10%)
Unemployed	20 (16.66%)
Type of cancer, n (%)	
Non-invasive	44 (36.66%)
Invasive	58 (48.33%)
Metastatic	18 (15%)

We achieved the following results by grouping the subjects according to their domain scores: All the subjects were depressed, and none of them had slight or mild depression. The majority, 71 (59.16%) subjects, experienced moderate depression, followed by severe depression, 49 (40.83%) subjects. All subjects had anxiety, with 35 (29.16%) having mild anxiety, 50 (41.66%) having moderate anxiety, and 35 (29.16%) having severe anxiety. All the subjects had somatic symptoms, but none were severe. A total of 22 (18.33%) subjects had minor somatic symptoms, 54 (45%) had mild somatic symptoms, and 44 (36.66%) had strong somatic symptoms. Suicidal ideation was reported by 79 (65.83%) subjects, with 50 (41.66%) having slight ideation and 29 (24.16%) having mild ideation, while 41 (34.16%) did not have any suicidal ideation. Sleep was another part of life that was impacted, with more than half of the subjects, 65 (54.16%), having mild sleep problems, 46 (38.33%) having moderate sleep disorders, and nine (7.50%) having severe sleep disorders. All of these facts are summarized in Table 2.

**Table 2:** DSM level 1 scores by domain n: Number of subjects; DSM: Diagnostic and Statistical Manual of Mental Disorders, Fifth Edition. "-" indicates no subjects.

Domain	Score of domain				
	0, n (%)	1, n (%)	2, n (%)	3, n (%)	4, n (%)
Domain I – Depression	-	-	-	71 (59.16%)	49 (40.83%)
Domain IV – Anxiety	-	-	35 (29.16%)	50 (41.66%)	35 (29.16%)
Domain V – Somatic symptoms	-	22 (18.33%)	54 (45%)	44 (36.66%)	-
Domain VI – Suicidal ideation	41 (34.16%)	50 (41.66%)	29 (24.16%)	-	-
Domain VIII – Sleep disorders	-	-	65 (54.16%)	46 (38.33%)	9 (7.50%)

Following that, we split the participants into three groups based on their type of cancer. We next computed descriptive statistics for each DSM level 1 domain score and WHODAS 2.0 test score in each group. The results are shown in Table 3.

**Table 3:** Descriptive statistics grouped by the type of cancer SD: Standard deviation; WHODAS: World Health Organization Disability Assessment Schedule.

Type of cancer		Domain I – Depression	Domain IV – Anxiety	Domain V – Somatic symptoms	Domain VI – Suicidal ideation	Domain VIII – Sleep disorders	WHODAS 2.0 score
Non-invasive	Mean	3.32	2.73	1.89	0.57	2.61	69.36
	Median	3.00	3.00	2.00	0.50	2.50	70.00
	SD	0.471	0.758	0.754	0.625	0.689	7.927
	Minimum	3	2	1	0	2	52
	Maximum	4	4	3	2	4	79
Invasive	Mean	3.43	3.12	2.24	1.07	2.43	95.03
	Median	3.00	3.00	2.00	1.00	2.00	94.50
	SD	0.500	0.751	0.657	0.792	0.596	15.994
	Minimum	3	2	1	0	2	63
	Maximum	4	4	3	2	4	126
With metastases	Mean	3.56	3.28	2.72	1.17	2.67	118.72
	Median	4.00	3.00	3.00	1.00	3.00	124.00
	SD	0.511	0.669	0.461	0.707	0.594	20.254
	Minimum	3	2	2	0	2	84
	Maximum	4	4	3	2	4	145

We applied the Kruskal-Wallis test, followed by the Mann-Whitney U test with Bonferroni correction in the case of a significant value (p < 0.05) in the Kruskal-Wallis test. By comparing the three groups (regarding the severity of the diagnosis) of subjects, we obtained the following results.

There were no statistically significant differences between the three groups regarding domain I (depression) and domain VIII (sleep disorders). There was a statistically significant difference in domain IV (anxiety) scores across the three groups (p = 0.009), particularly between the non-invasive and invasive groups (p = 0.031) and between the non-invasive and metastatic groups (p = 0.031). Subjects with non-invasive cancer are less likely to experience anxiety than those with aggressive or metastatic cancer. Subjects with invasive cancer have the same anxiety as those with metastases.

Between the scores of domain V (somatic symptoms), there was a significant difference between the groups (p < 0,0001), more specifically between the non-invasive group and the metastatic group and between the invasive group and the metastatic group. Somatic symptoms are less pronounced in non-invasive and invasive subjects than in the ones with metastases, while there is no difference between the non-invasive and invasive groups.

The scores of domain VI (suicidal ideation) differed significantly between the groups (p = 0.001), notably between the non-invasive and invasive groups as well as between the non-invasive and metastatic groups. Suicidal ideation is comparable in the invasive and metastatic groups, although the non-invasive group has lower levels.

When we evaluated the WHODAS 2.0 disability scores, we found a significant difference between the groups (p < 0.001). When we compared each group pairwise, we discovered substantial differences. Subjects with non-invasive cancer had the lowest WHODAS 2.0 score, followed by the invasive group, while metastases had the highest score. All the results are summarized in Table 4.

**Table 4:** Kruskal-Wallis test and Mann-Whitney U test with Bonferroni correction test results comparing the subjects’ groups 0-1 (p): p-value of pairwise comparison (applying Mann-Whitney U test with Bonferroni correction) between group 0 (non-invasive cancer) and group 1 (invasive cancer). 0-2 (p): p-value of pairwise comparison (applying Mann-Whitney U test with Bonferroni correction) between group 0 (non-invasive cancer) and group 2 (metastatic cancer). 1-2 (p): p-value of pairwise comparison (applying Mann-Whitney U test with Bonferroni correction) between group 1 (invasive cancer) and group 2 (metastatic cancer). Kruskal-Wallis test (p): p-value of the Kruskal-Wallis test comparing the three groups. WHODAS: World Health Organization Disability Assessment Schedule.

	0-1 (p)	0-2 (p)	1-2 (p)	Kruskal-Wallis test (p)
Domain I – Depression	-	-	-	0.203
Domain IV – Anxiety	0.031	0.031	1.000	0.009
Domain V – Somatic symptoms	0.059	<0.001	0.031	<0.001
Domain VI – Suicidal ideation	0.003	0.014	1.000	0.001
Domain VIII – Sleep disorders	-	-	-	0.195
WHODAS 2.0 score	<0.001	<0.001	0.014	<0.001

## Discussion

Patients with breast cancer have a heavy psychological burden due to the particularity of the disease and are most of the time accompanied by various mental problems, depression, and anxiety symptoms being particularly common. Some studies attest to the fact that in addition to depression and anxiety, other symptoms such as insomnia, somatization symptoms, and many others can appear [21]. The prevalence of psychiatric disorders in breast cancer patients is estimated in some studies to be 47%, of which 68% have mild/moderate anxiety and depression, 13% have major depression, and 8% have delirium [22]. Patients diagnosed with breast cancer in advanced stages (pre-terminal and terminal) are a particularly vulnerable group; 25% of all neoplastic patients have severe depressive symptoms, and this prevalence increases to 77% in those in advanced evolutionary stages [22]. Our study showed comparable results, with all our patients suffering from anxiety and depression in varying degrees, particularly moderate and severe.

Studies show that approximately 25% of breast cancer patients have a depressed mood, and 10%-15% of patients are diagnosed with major depression during oncological treatment [23]. Risk factors regarding the development of depressive episodes are young age, advanced stage of the disease, alcohol, and poor social support network [24]. In 2015, a study was conducted on a group of 100 patients diagnosed with breast cancer in different stages to establish the impact coefficient on the disease and the psychological implications of the somatic condition. The results found that 38% of them presented symptomatology characteristics of a moderate depressive episode, 29% had severe depression, 20% had mild depression, and 4% were asymptomatic [24]. Recent studies have shown that psychiatric comorbidity is associated with impaired quality of life, increased symptom load, and decreased adherence to therapy [25].

Patients with neoplastic pathology associated with severe pain have a two times higher risk of developing psychopathological disorders compared to those without pain or with low-intensity, inconstant pain [26]. We discovered substantial variations in somatic symptoms between the metastatic and non-invasive/invasive groups, with participants who had metastases scoring higher than those without.

Long-term patient surveillance studies have described the association between depressive symptoms, the predisposition to develop malignant diseases, and the survival rate following the diagnosis of breast cancer [27,28]. While comparing the grade of depression between the types of cancer, we found no statistical difference between them, although all patients suffered from moderate and severe depression. By assessing them again after the start of the treatment, we may find differences in the severity of depression as more patients will get over the depressive episode.

Even if the symptoms of anxiety are comparable to those of depression, it is not always identified and treated appropriately. Most of the time, it is treated when it is secondary to depression [29]. The risk of patients developing a psychopathological episode after being diagnosed with breast cancer increases with the severity of cancer, aggressive treatments, limited functionality, inadequate social support network, or a history of mental illness [30]. When we compared the three groups, we found significant differences in the anxiety levels between the non-invasive and the other two groups, but no difference was observed between the invasive and metastatic groups.

About 26% of cancer patients reported deterioration of physical work capacity, and 19% of them stated that these problems persist between two and six years after diagnosis. Therefore, cancer is a major cause of absenteeism, unemployment, and early retirement [30,31]. In our study, we compared the WHODAS disability score between the three groups, obtaining higher scores as the severity of the cancer increased. Physical work capacity is also affected as the WHODAS score increases, with work-related questions being part of the questionnaire.

Numerous studies have highlighted the unfavorable situation faced by the patient's families and the difficulties they face when caring for the patients. Patient care can be overwhelming for many family members, so the social support network is destabilized, the patient feels hopeless, and psychiatric symptoms increase. The literature shows clear evidence of social isolation and decreased interaction [32]. This aspect is also reflected in the WHODAS score, which directly increases with the severity of the cancer in our patients.

The impact of breast cancer diagnosis and the implications of aggressive treatment affect patients' daily activities, independence, and autonomy. Addiction and loss of identity cause anguish and multiple fears, thus affecting emotional balance and quality of life [33]. Another question on the WHODAS disability score is related to addictions, which, in turn, can justify why patients with more severe cancers can develop addictions.

Strengths, limitations, and future directions

The study sheds light on the psychiatric ramifications of an oncological diagnosis, which are often overlooked because treatment focuses on the oncological diagnosis rather than the psychological implications.

Another strength of the study is the assessment of the patient's disability, which might have an impact on his daily life and social interactions, possibly leading to a vicious cycle that worsens the psychiatric symptoms. Addressing this issue as a healthcare professional has the potential to significantly enhance the patients' somatic and psychological outcomes.

The current study had some limitations that deserve to be addressed by future researchers. One of the limitations was sending the tests online to the subjects, thus having the subjects self-administer the questionnaires.

Another limitation that can benefit from a future improvement to the study could be a second assessment of the test after a second appointment with the oncologist or after beginning treatment to address all of the subjects' questions about the disease following possible self-information from potentially untrustworthy internet sources.

Another possible future direction for an analogous study would be to investigate other types of malignancies that affect both men and women or exclusively men (such as prostate cancer) to see whether different psychological implications depend on the affected organ system or gender.

## Conclusions

WHODAS 2.0 is a useful and appropriate tool for assessing disability and functioning from the perspective of the breast cancer patient. If the specialists are informed in as much detail as possible about the daily limitations of the patients, rehabilitation and treatment measures can be applied to improve the quality of life. It is necessary to know if the patients will be able to work and carry out their daily routines to fulfill their roles at home, at work, or in other social fields. In our study, patients with non-invasive cancer had the lowest WHODAS 2.0 score, followed by the invasive group, while metastases had the highest score.

Screening for psychiatric symptoms (level 1 test) offers an opportunity to improve the quality of care of cancer patients, thereby improving treatment adherence and clinical outcomes. We found statistical differences between the scores of the non-invasive group and the invasive group or metastatic group regarding anxiety or suicidal intentions, while there was no difference between the invasive and metastatic groups in these two domains. Identifying a core set of symptoms and/or related domains is absolutely necessary because a personality prone to developing mental illness is an obstacle to adherence to treatment.
